# p53-Independent regulation of p21Waf1/Cip1 expression and senescence by PRMT6

**DOI:** 10.1093/nar/gks858

**Published:** 2012-09-16

**Authors:** Sameer Phalke, Slim Mzoughi, Marco Bezzi, Nancy Jennifer, Wei Chuen Mok, Diana H. P. Low, Aye Aye Thike, Vladimir A. Kuznetsov, Puay Hoon Tan, P. Mathijs Voorhoeve, Ernesto Guccione

**Affiliations:** ^1^Division of Cancer Genetics and Therapeutics, Institute of Molecular and Cell Biology (IMCB), A*STAR (Agency for Science, Technology and Research), Singapore 138673, ^2^Department of Biochemistry, Yong Loo Lin School of Medicine, National University of Singapore, ^3^Division of Genome and Gene Expression Data Analysis, BioInformatics Institute (BII), A*STAR (Agency for Science, Technology and Research), Singapore 138671, ^4^Department of Pathology, Singapore General Hospital, Outram Road, Singapore 169608, ^5^School of Computer Engineering, Nanyang Technological University, N4-02a-27, 50 Nanyang Avenue, Singapore 639798 and ^6^Cancer and Stem Cell Biology Program, Duke NUS, 8 College Road, Singapore 169857

## Abstract

p21 is a potent cyclin-dependent kinase inhibitor that plays a role in promoting G1 cell cycle arrest and cellular senescence. Consistent with this role, p21 is a downstream target of several tumour suppressors and oncogenes, and it is downregulated in the majority of tumours, including breast cancer. Here, we report that protein arginine methyltransferase 6 (PRMT6), a type I PRMT known to act as a transcriptional cofactor, directly represses the p21 promoter. PRMT6 knock-down (KD) results in a p21 derepression in breast cancer cells, which is p53-independent, and leads to cell cycle arrest, cellular senescence and reduced growth in soft agar assays and in severe combined immunodeficiency (SCID) mice for all the cancer lines examined. We finally show that bypassing the p21-mediated arrest rescues PRMT6 KD cells from senescence, and it restores their ability to grow on soft agar. We conclude that PRMT6 acts as an oncogene in breast cancer cells, promoting growth and preventing senescence, making it an attractive target for cancer therapy.

## INTRODUCTION

Histone methylation, occurring on arginine (R) and lysine (K) residues, is a key post-translational modification (PTM) mediating epigenetic control of transcription (1–5). Two key amino acids involved in transcriptional regulation are located on the N-terminal tail of histone H3: arginine at position 2 (H3R2) and lysine at position 4 (H3K4). Several groups, including our own, have described the negative cross-talk between the asymmetric dimethylation of arginine 2 (H3R2me2a), by protein arginine methyltransferase 6 (PRMT6), and the tri-methylation of lysine 4 (H3K4me3), by mixed lineage leukaemia 1–4 and Set1a/b (1,2,6,7). Trimethylation of lysine 4 on histone H3 (H3K4me3) is a PTM present at active and poised promoters, whereas it is depleted at facultative and constitutive heterochromatic sites (4,5,8,9). H3R2m2a is instead present at repressed promoters (1,2,6).

PRMT6 is a member of the PRMT family. PRMTs are enzymes that methylate proteins, including histones, on arginine residues (10). Members of this family bind to chromatin, act as transcriptional co-activators or co-repressors and are deregulated in diverse cancer types (11–13).

Breast cancer is the most common malignancy and a leading cause of cancer death among women in USA (14). The inefficacy in breast cancer treatment originates from our poor understanding of the disease because of its complex phenotypes. Based on the molecular phenotype, breast cancer can be divided largely into luminal subtypes, which show relatively good responsiveness to treatment (15,16), and the basal-like subtypes, which represents 75% of the triple negative [estrogen receptor (ER)^−^, progesterone receptor (PR)^−^, Her2^−^], aggressive and invasive population (17) and 15–20% of breast cancers (18–20). Generally, poor prognosis associates with the basal-like subtype and with inactivation of p53 pathways (21).

Different subtypes are associated with distinct chromatin structures and epigenetic signatures, and it is, thus, of central importance to define which transcription factors or epigenetic regulators underpin such changes to design efficient therapies. Here, we focus our investigation on PRMT6 and on its deregulation in breast cancer. Despite its overexpression in a variety of cancer tissues (13), a causal link demonstrating that the upregulated enzymatic activity of PRMT6 is essential for malignant transformation has not been established. Similarly, the consequence of downregulating PRMT6 activity in cancer cells, and the identification of relevant downstream targets, with the exception of the inhibitor of cell migration thrombospondin 1 (22), has not been fully elucidated, hampering its potential exploitation as a drug target in oncology.

We show that PRMT6 acts as a transcriptional co-repressor by directly biding to the p21 promoter, where it methylates H3R2me2a. This correlates with the presence of other repressive marks, such as H3K27me3, and the absence of H3K4me3 and H3 acetylation. On PRMT6 depletion, p21 levels are increased, and this is, importantly, a p53-independent mechanism. The consequences of p21 upregulation are as follows: cell cycle arrest, cellular senescence and reduced growth in soft agar assays and in severe combined immunodeficiency (SCID) mice. Finally, we show that reducing p21 levels by a short hairpin RNA (shRNA) is sufficient to bypass the inhibitory effects of PRMT6 knock-down (KD) on cell proliferation and anchorage independent growth. We conclude that PRMT6 promotes cell growth and prevents senescence, thus, making it potential target for cancer therapy.

## MATERIALS AND METHODS

### Cell culture

Human breast cancer cell line MCF7, MDA-MB-231, SK-BR-3, MDA-MB-468, MCF10A and BT474 were obtained from ATCC and were propagated according to ATCC data sheets.

### Vectors, transfections, infections

pLKO Mission (Sigma) lentiviral vectors were used for PRMT6 KD in different cell lines. Two independent KD shRNA constructs (sh-1 and sh-2) were used, the sequences of the shRNA are sh-1: CACCGGCATTCTGAGCATCTT, sh-2: CGCATACTTCTGCGCTACAAA; a scrambled shRNA (sh-C) was used as a control. pSuper retroviral vector, with a short hairpin (sh-p21: GACCATGTGGACCTGTCAC) targeting p21, was used to KD p21.

### Real-time quantitative reverse-transcriptase polymerase chain reaction

Total RNA was isolated from the cells using RNeasy Mini kit (Quiagen). One microgram RNA was used to prepare cyclic DNA (cDNA), using Vilo cDNA kit (Invitrogen). The cDNA prepared was subjected to quantitative reverse-transcriptase polymerase chain reaction (qRT-PCR), using SYBR Green PCR Supermix from Invitrogen in an ABI PRISM 7500 sequence detection system with 96-well module and automation accessories (Applied Biosystems). Expressions were normalized to actin or glyceraldehyde-3-phosphate dehydrogenase (GAPDH). Each sample was analysed in triplicate, and representative data sets are shown. Primer sequences are given as follows: PRMT6 (forward: ctgctgcgctacaaagtgg, reverse: agaaaaggcaacgctcagtc), p21 (forward: ggcctggactgttttctctcg, reverse: gagaaacgggaaccaggacac), p16 (forward: gagcagcatggagccttc, reverse: cctccgaccgtaactattcg), p53 (forward: gctgctcagatagcgatggtct, reverse: catccaaatactccacacgcaa), actin (forward: cgtcttcccctccatcgt, reverse: gaaggtgtggtgccagattt), GAPDH (forward: gaaggtgaaggtcggagtc, reverse: gaagatggtgatgggatttc).

### Western blotting and antibodies

For western blotting, cells were lysed in 1× Laemmli buffer and sonicated with three short pulses before loading on sodium dodecyl sulphate polyacrylimide gels (SDS–PAGE). The proteins were resolved on 8–15% SDS–PAGE and subsequently transferred on nitrocellulose or PVDF membranes. The membranes were incubated overnight with primary antibodies against PRMT6 (Abcam, Cat No# ab47244-200), β-actin (Santa Cruz, Cat No# sc47778), p21 (Cell Signalling, Cat No# 2947), p53 (DO-1, Santa Cruz Cat No# sc126), phospho p53 (Cell Signalling, Cat No# 9284). Horseradish peroxidase (HRP) tagged secondary antibodies were used from Santa Cruz. The antibodies used for chromatin immunoprecipitation (ChIP) were the following: H3K27me3 (Millipore, Cat No# 07-449), H3Ac (Millipore Cat No# 06-599), H3 (Abcam Cat No# ab1791), H3K4me3 (Diagenode Cat No# pAb-030-50). H3R2me2a antibody: four rabbits were immunized with keyhole limpet haemocyanin-coupled peptides encompassing the first 10 amino acids of histone H3, asymmetrically modified on R2 (the peptide was purchased from Mimotopes—Clayton, Victoria, Australia—and the immunization was done at Millipore—Billerica, MA, USA). Affinity-purified or crude sera were used for western blotting and ChIP experiments.

### Chromatin immunoprecipitation

MCF7 cells stably expressing scrambled (sh-c) or shRNAs targeting PRMT6 (sh-1 and sh-2) were processed for qChIP following our original protocol (23), with further modifications. Formaldehyde (37%) was added to the culture medium to a final concentration of 1%. Cross-linking was allowed to continue for 10 min at room temperature and was stopped by addition of glycine (0.125 M as final concentration), followed by an additional incubation of 5 min. Fixed cells were washed twice with phosphate buffered saline (PBS) and harvested in SDS buffer (50 mM tris at pH 8.1, 0.5% SDS, 100 mM NaCl, 5 mM ethylenediaminetetraacetic acid (EDTA) and protease inhibitors). Cells were pelleted by centrifugation and were resuspended in 3 ml of FA lysis buffer (50 mM tris–HCl pH 7.5, 150 mM NaCl, 1 mM EDTA, 1% triton X-100). Cells were disrupted by 5–7 pulses (30 s each one) of sonication with a tapered microtip (6.5 mm) in a Branson digital sonifier 250 D, at a power setting of 30%, yielding genomic DNA fragments with a bulk size of 300–500 bp. For each immunoprecipitation, 1 ml of diluted lysate (5 × 107 cells/ml) was pre-cleared, by addition of 300 μl-blocked protein A or G beads [50% slurry protein A Sepharose from Amersham or Rec-protein G-Sepharose 4B ZYMED from Invitrogen, 0.5 mg/ml fatty acid free bovine serum albumin from Sigma and 0.2 mg/ml salmon sperm single-stranded DNA (ssDNA) in Tris EDTA]. Samples were immunoprecipitated overnight at 4°C with antibodies specific for specific proteins or modified histones. Samples were incubated with protein G or A Sepharose beads for 3 h, followed by series of high stringency washes. The bound DNA was reverse cross-linked and purified using 1:10 w/v Chelex beads. Eluted DNA was used as template for real-time qPCR. Primers spanning up to 5 kb upstream of p21 start site were analysed for the enrichment of PRMT6 and histone modifications. Primer sequences are available on request.

### Soft agar assay

To evaluate the anchorage independent growth potential of PRMT6 KD and/or p21 KD cells, 50 000 MCF7 and MDA-MB-231 cells infected with scrambled control (sh-c) or PRMT6 KD constructs (sh-1/sh-2) and/or sh-p21 were resuspended in a 0.8% agarose (Sigma Aldrich) medium and were laid on a bottom agarose layer containing 1.5% agarose. Once solidified, the plates were incubated at 37°C for ∼3 weeks, fresh medium was added to the plates in every 2–3 days. To visualize and count the colonies, methylthiazoletetrazolium (MTT) (Calbiochem, Cat No# 475989) was added on the colonies to a final concentration of 5 mg/ml in Dulbecco’s modified Eagle’s medium on top of the plate, and the plates were incubated for 30–60 min at 37°C in dark. The stained plates were then scanned on Epson Perfection 4490 scanner at 800 dpi. Acquired images were then quantified on ImageJ software. At least three independent experiments were performed in triplicate, error bars represents the variations in triplicates from each experiment.

### Senescence-associated acidic β-galactosidase staining assay

Cells were grown in to monolayer, washed twice with ice cold PBS, fixed with 2% formaldehyde and 0.2% gluteraldehyde for 5 min at room temperature. Fixed cells were washed twice with PBS followed by incubation with staining solution containing 1 mg/ml X-gal, 2 mM MgCl_2_, 5 mM each of potasium ferricynide and potasium ferrocynide in 40 mM citric acid/sodium phosphate buffer for 2–8 h at 37°C in dark. After incubation, the cells were washed with PBS and stored at 4°C until analysis. Images were acquired with Olympus microscope and senescence-associated acidic β-galactosidase staining (SABG), positive cells were counted on Adobe Photoshope CS4 software. At least three independent experiments were performed, and counting was done on three randomly selected fields from each plate. Error bars of different experiments are indicated.

### Tissue microarray

In-house generated polyclonal antibody against the full-length recombinant human PRMT6 was used at 1:5000 dilution. Epitope retrieval: Citrate buffer 10 mM 6 pH, pressure cooker (121°C) 45 min. Peroxidase block: 3% hydrogen peroxide 30 min. Serum block:10% goat serum in Tris Buffered Saline and Tween 20 (TBST) 60 min. Primary antibody incubation: 60 min at room temperature. Secondary antibody: DAKO anti-rabbit HRP polymer Cat No# K4003 (undiluted, incubated for 30 min). Diaminobenzidine (DAB) kit: DAKO Cat No# K3468.

The analysis was carried out on 215 cases of breast cancer and normal breast epithelium [tissue microarray (TMA) BR2001 and BR802 from US Biomax, Inc.]. The staining intensity and % of positive tumour cells were scored. For matched control tissues (TMA BR802), we used the threshold of ≥1% positively stained tumour cells to define PRMT6 positivity. On the larger sample of tumour samples (TMA BR2001), PRMT6 positivity was defined using the cut-off value of at least 10% of tumour cells expressed PRMT6 regardless of staining intensity.

### *In vivo* tumour growth analysis

MCF7 and MDA-MB231 breast carcinoma cells, transfected with constructs depleting PRMT6 (sh-2) or scramble control sh-c, were resuspended in PBS at a concentration of 5 × 10^6^/100 μl and 1 × 10^6^/100 μl. Hundred microlitres of this suspension was orthotopically injected into the mammary fat pad of 6–8-week-old SCID mice, after anesthetizing the animals by immunoprecipitation injection of avertin (300 mg/kg). A small incision is performed through the skin and subcutaneous tissue in the abdominal area to expose the fourth mammary fat pads. Mice were stitched with 3–0 braided absorbable sutures. After 2–12 weeks, the sizes of the tumours were monitored. Mice were aged and tumour development was evaluated. All animals that seemed to be moribund, cachectic or unable to obtain food or water were euthanized.

### Statistical analysis

Statistical analysis for cell cycle distribution and soft agar assay and X-gal staining were performed using Pearson’s χ^2^ analysis and student’s one-tailed *t*-test, respectively. A resulting *P*-value of 0.05 or lower was considered as significant.

## RESULTS

### PRMT6 is overexpressed in breast cancer

**Figure 1.**
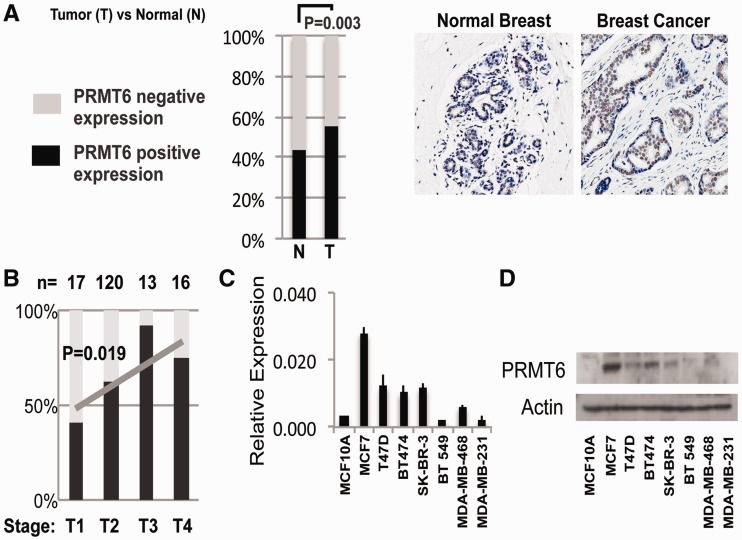
PRMT6 is overexpressed in breast tumours. (**A**) PRMT6 is overexpressed in tumours as compared with the normal tissue based on the analysis of 37 matched control cases. On the right, representative stainings from the TMA are shown as follows: normal breast epithelium (left) and breast carcinoma (right). (**B**) PRMT6 overexpression is positively correlated (*P* = 0.019) with tumour stage. Analysis is based on 166 breast cancer samples. (**C**) PRMT6 mRNA expression in a panel of breast cancer cell lines. (**D**) PRMT6 protein levels in a panel of breast cancer cell lines.

Overexpression of PRMT1 and PRMT6 has been previously reported in various cancer types (13). To study the role of PRMT6 in breast cancer, we raised a PRMT6 specific antibody (Supplementary Figure S1) and examined its expression levels in 37 tumour samples and matched control tissues. We found a significant overexpression of PRMT6 in 54.1% of tumour samples as compared with 43.9% in normal breast epithelium (Figure 1A, examples are shown on the right). This observation prompted us to analyse PRMT6 expression in a larger set of cancer samples. We stained an additional 215 samples and found 58.6% of tumours expressing high levels of PRMT6. Interestingly, for those samples for which we had clinical information regarding the tumour stage (*n* = 166), we observed positive correlation (*P* = 0.019) between PRMT6 up-regulation and tumour stage (Figure 1B), suggesting that more aggressive tumours might benefit from PRMT6 overexpression. With the aim to mechanistically dissect the putative role of PRMT6 in the aetiology of breast cancer, we then switched to cancer cell lines. Similar to our results from tumours and normal tissue, we found PRMT6 to be overexpressed at the RNA (Figure 1C) and protein (Figure 1D) levels in cancer lines as opposed to MCF10A normal breast epithelial cells.

### Loss of PRMT6 induces cell cycle arrest and inhibition of tumour growth *in vitro* and *in vivo*

**Figure 2.**
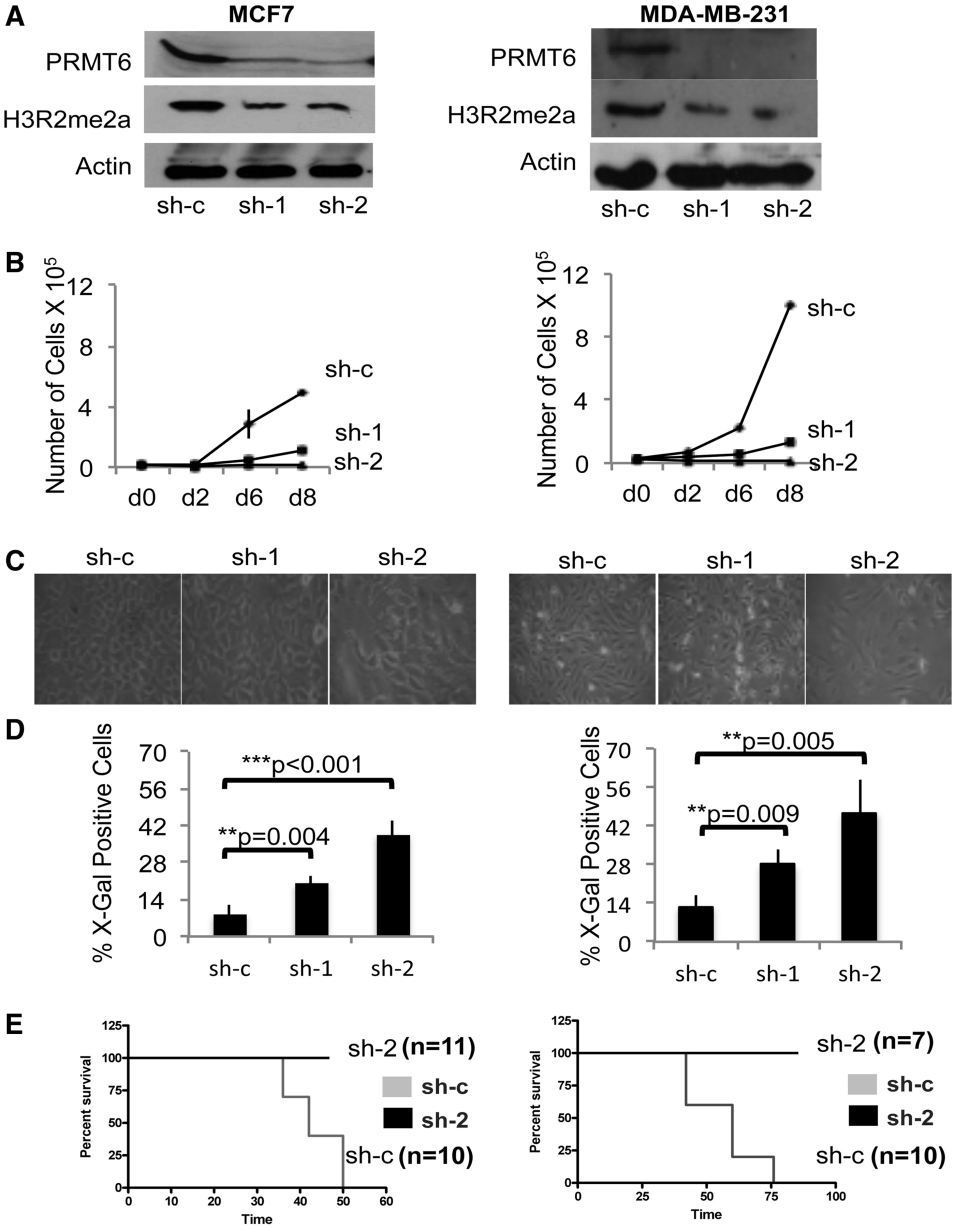
Loss of PRMT6 induces cell cycle arrest and inhibition of tumour growth *in vitro* and *in vivo.* (**A**) Two independent shRNA constructs against PRMT6 (sh-1 and sh-2) efficiently KD the protein levels along with the associated H3R2me2a arginine methylation mark as compared with the control shRNA (sh-c). The experiment is performed in MCF7 (left panel) and MDA-MB-231 (right panel) cells. (**B**) Both shRNA reduce proliferation as compared with the control in both the cells lines. (**C**) PRMT6 KD induces morphological changes typical of senescent cells, appearing flat and (**D**) staining for SABG. (**E**) Orthotopic injections of MCF7 (left panel) and MDA-MB-231 (right panel) cells depleted of PRMT6 (sh-2, black line) in SCID mice mammary fat pad completely inhibits tumour formation as compared with the mice injected with control cells (sh-c, grey line). Number of females injected for each condition are indicated on the side of each graph.

We next sought to determine whether PRMT6 expression was required for growth and tumorigenicity of breast cancer cell lines. We generated lentiviral vectors stably expressing specific shRNAs against PRMT6 (sh-1, sh-2) and analysed the effects of PRMT6 KD in MCF7 (luminal, ER^+^ cells expressing wild-type p53), and MDA-MB-231 cells (basal, triple negative and mutant p53). A KD efficiency >80% could be achieved at the RNA and protein levels (Figure 2A and Supplementary Figure S2). PRMT6 KD in both cell lines inhibits growth (Figure 2B) and specifically leads to a reduction of cells in S phase (Supplementary Figure S3). Both sh-1 and sh-2 cells show a clear change in morphology, with cells looking bigger and flatter as compared with the scrambled control shRNA (sh-c) (Figure 2C). As the morphology and the cell cycle arrest was reminiscent of cellular senescence, we confirmed this by SABG on the PRMT6 KD and control cells (Figure 1D). To finally check whether the induction of a growth arrest and senescence by PRMT6 depletion was relevant *in vivo*, we injected immunocompromised SCID mice with equal amounts of either MCF7 or MDA-MB-231 cells tranduced with PRMT6 KD (sh-2) or scramble control vectors (*n* = 10 for sh-c; *n* = 11 and *n* = 7 for sh-2 in MCF7 and MDA-MB-231, respectively). MCF7 and MDA-MB-231 sh-c control cells generated primary tumour within 6–8 weeks. Consistently with our *in vitro* data, PRMT6 sh-2 KD cells did not form primary tumours in any of the injected animals (Figure 2E).

### PRMT6 directly represses the p21 promoter

**Figure 3.**
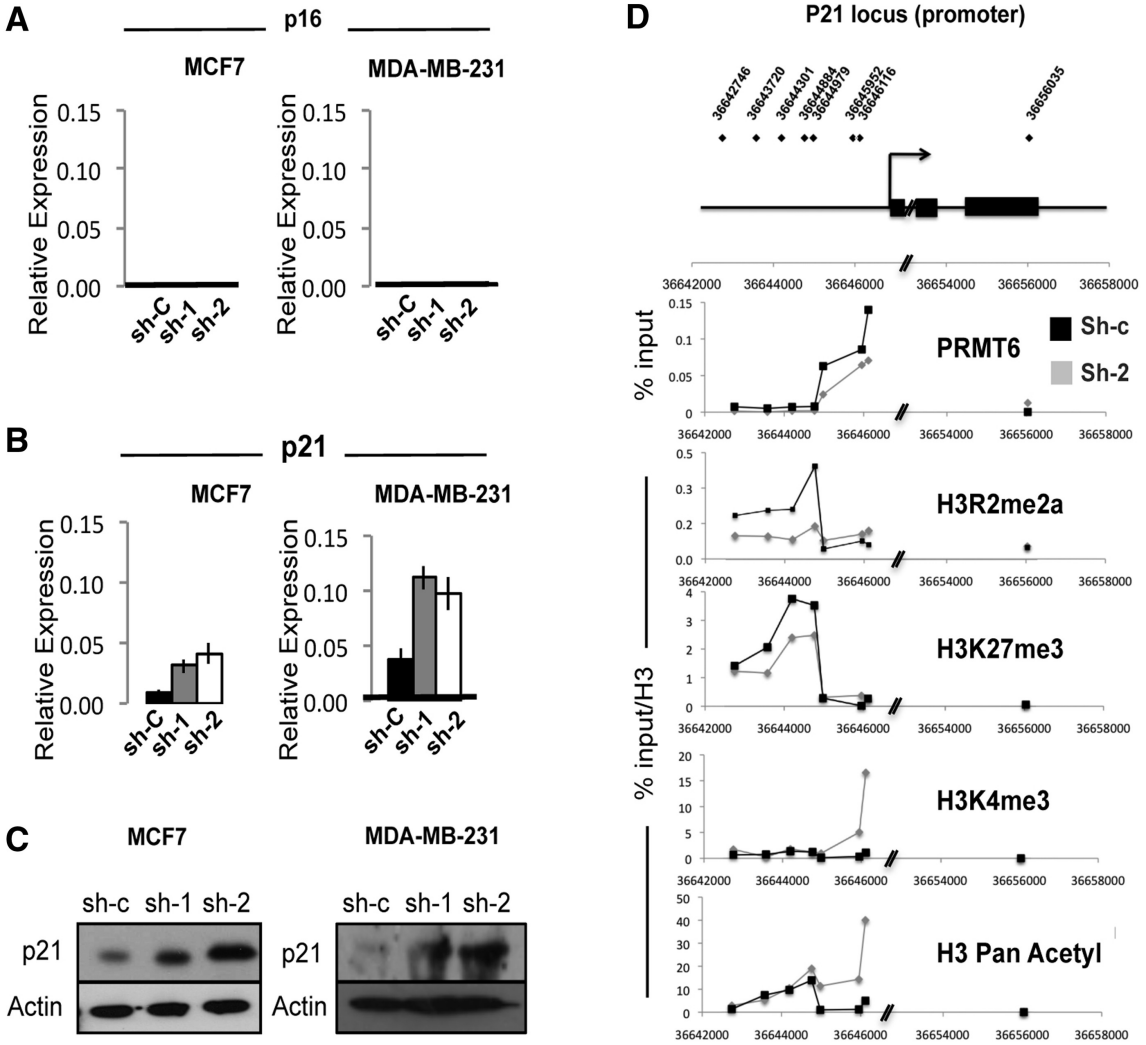
PRMT6 directly represses the p21 promoter. qPCR analysis of cyclin inhibitors (**A**) p16 and (**B**) p21 in PRMT6 depleted (sh-1 and sh-2) or control (sh-c) MCF7 and MDA-MB-231 cells. (**C**) Immunoblot analysis of p21 protein levels in PRMT6 depleted (sh-1 and sh-2) or control (sh-c) MCF7 and MDA-MB-231 cells. (**D**) Chromatin immunoprecipitation in MCF7 cells using the antibody indicated on each panel. Data are shown as the % of input DNA for PRMT6 and as % of input DNA normalized to total H3 (% input/H3) for the histone PTMs. The p21 promoter in MCF7 cells is enriched for inactive- (H3R2me2a and H3K27me3) and depleted for active- (H3K4me3 and H3acetyl) chromatin marks in control cells as compared with PRMT6 depleted ones (sh-2, grey lines). Experiment was repeated four times, and a representative plot is shown.

We hypothesized that as PRMT6 is required for epigenetic silencing, the growth arrest we observed *in vitro* and *in vivo* could be mediated by the deregulated expression of crucial PRMT6 target genes. Therefore, to understand the molecular basis of the growth arrest induced by PRMT6 KD in breast cancer cells, we focused our attention on two key mediator of cellular senescence as follows: p16^INK4A^ and CDKN1A/p21/Waf1/cip1 (p21). Although p16 levels did not change in either MCF7 [the p16 locus is deleted in these cells (24)] or MDA-MB-231 (wt p16) (Figure 3A), we observed a striking upregulation of p21 messenger RNA (mRNA) (Figure 3B) and protein levels (Figure 3C).

PRMT6 is a known transcriptional repressor, and the methylation of H3R2me2a has been shown to counter-correlate with H3K4me3 and transcriptional activation. Therefore, we asked whether PRMT6 could directly regulate the expression of the p21 locus. To this end, we performed chromatin immunoprecipitation using specific antibodies for PRMT6, H3R2me2a, H3K27me3, H3K4me3 and acetylated H3 antibodies in MCF7 to assess the binding of PRMT6 and changes in the chromatin landscape in the upstream regulatory regions of p21 on PRMT6 modulation. Our data showed a reproducible binding of PRMT6 upstream of the p21 locus (sh-c, black line), which was significantly reduced on PRMT6 KD (sh-2, grey line) (Figure 3D). In MCF7 cells, the p21 promoter is bound by PRMT6, methylated on H3R2me2a and H3K27me3, and p21 is expressed at low levels (Figure 3B–D). On PRMT6 KD, the repressive H3R2me2a and H3K27me3 marks are lost and substituted by the activation marks (H3K4me3 and H3 pan acetyl). In summary, our data demonstrate the direct transcriptional repressor function of PRMT6 on the p21 promoter.

### PRMT6 mediated p21 regulation is independent of p53

To further confirm that p21 regulation by PRMT6 is indeed p53 independent, we first checked the levels of p53 in MCF7 PRMT6 KD cells. We did not see any significant changes in p53 levels at RNA and protein levels (Supplementary Figure S4A and B). We then KD PRMT6 in MCF7, followed by treatment with the p53 inhibitor pifithrin (PFT) to inhibit p53 activation (25). As a control sample, we induced p53 using DNA damage inducing drug etoposide (ETP). ETP induced p53 stabilization and phosphorylation and upregulated p21 expression (Supplementary Figure S4C). Cells pre-treated with PFT showed a reduced p53 response on ETP treatment as demonstrated by reduced p53 phosphorylation and p21 induction. Importantly, PRMT6 KD led to p21 induction to similar levels irrespective of p53 inhibition by PFT (Supplementary Figure S4D), demonstrating that PRMT6 mediated p21 regulation is independent of p53.

As we observed that PRMT6 KD in p53-mutant MDA-MB-231 cells also results in a growth arrest, with p21 overexpression, we sought to confirm the link between PRMT6 and p21 in these cells and to extend these observations to additional cell lines that lack functional p53 (SK-BR-3 and MDA-MB-468). PRMT6 KD consistently led to p21 upregulation, cell cycle arrest and cellular senescence in all cell lines tested irrespective of p53 status (Supplementary Figure S5 and data not shown). These data suggest that inhibiting PRMT6 functions might be extremely relevant to reduce the tumorigenic potential of fast growing cancers, irrespective of their p53 status.

### Bypassing p21 overexpression rescues the PRMT6 depletion phenotype

**Figure 4.**
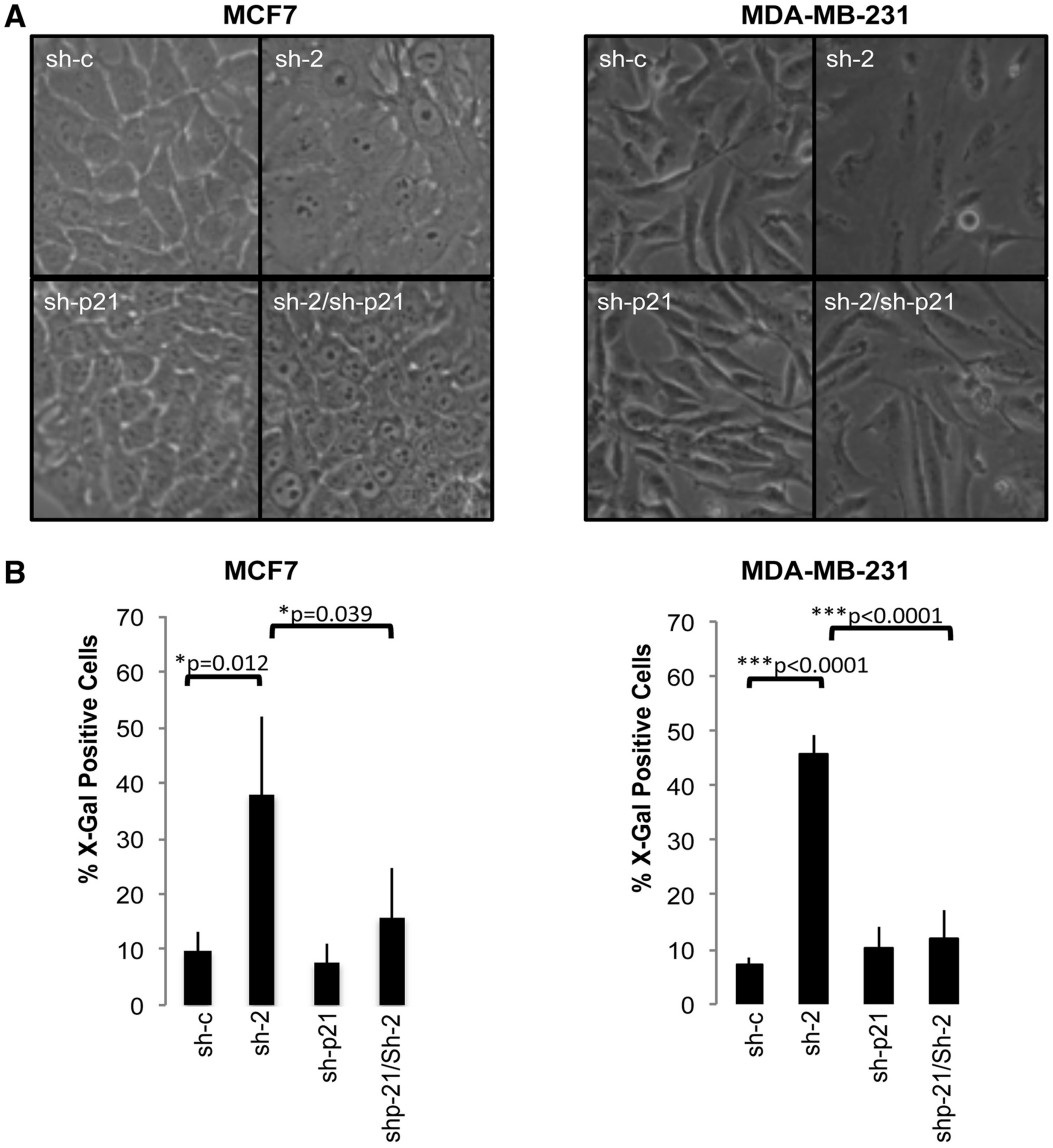
Bypassing p21 expression rescues PRMT6 depletion phenotype. (**A**) p21 KD (sh-p21) rescues the morphological changes (flat cell phenotype) induced by PRMT6 (sh-2) KD alone in MCF7 (left panel) and MDA-MB-231 (right panel). (**B**) Senescent cells are scored as assessed by SABG staining. p21 KD (sh-p21) is able to rescue PRMT6 KD induced senescence in both the cell lines. Error bars represent the variations in the number of cells staining positive for SABG in three independent experiments. *P*-values of the statistical significance are indicated.

**Figure 5.**
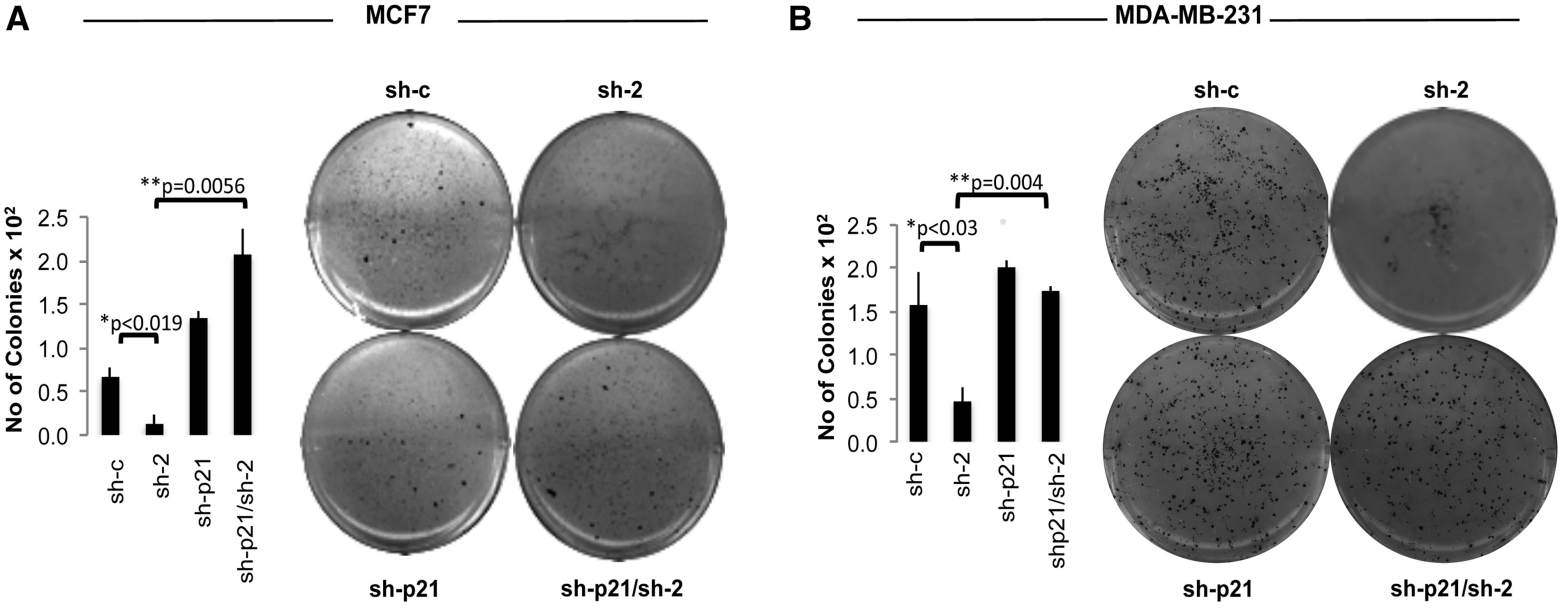
Depletion of p21 rescues the anchorage independent growth of PRMT6 depleted cells on soft agar. (**A**) Soft agar assay in MCF7 (left panel) and MDA-MB-231 (right panel), using PRMT6 KD (sh-2), p21 KD (sh-p21) or double PRMT6 and p21 KD (sh-p21/sh-2). (**B**) Quantification of the number of colonies in different conditions, error bars represents the variation in three independent experiments, *P*-values of the statistical significance are indicated.

Importantly, the consistent upregulation of p21, correlating with growth arrest in several cell lines, suggests that p21 could be one of the major mediators of the effects of PRMT6 KD on cell growth. To test this hypothesis, we asked whether p21 depletion could rescue the phenotypic effects driven by PRMT6 KD. When p21 was stably depleted using a specific shRNA expressing retrovirus (sh-p21), we observed a clear rescue of the morphological effects of PRMT6 KD (Figure 4A). Moreover, on double KD of PRMT6 and p21, cells did not undergo cell cycle arrest (Supplementary Figure S6) nor became senescent as assessed by SABG staining (Figure 4B). Finally, we tested these cells for their ability to grow in soft agar; although reducing PRMT6 levels lowers the number of colonies able to grow in an anchorage independent manner, the depletion of p21 completely reverses this effect in MCF7 (Figure 5A) and MDA-MB-231 cells (Figure 5B). Together these data show that PRMT6 depletion upregulate p21 levels, resulting in growth arrest and senescence, without requiring functional p53. Given that direct mutations of p21 are rare, even in tumours or cell lines that express wt p53 (such as MCF7), and are even rarer in cells bearing p53 mutations (26), upregulation of p21 is an interesting anti-tumour strategy. This would initially suggest that pharmacological inhibition of the enzymatic activity of PRMT6 (27) is an attractive strategy to target tumours.

## DISCUSSION

Here, we have characterized the direct repressive effect of PRMT6 on the p21 promoter. Based on the data presented, we can conclude that PRMT6 acts as an oncogene in breast cancer. PRMT6-inhibition leads to a reduced tumorigenic potential because of up-regulation of p21 (Figure 2), and it would, therefore, be an ideal drug target to slow down the growth of rapidly dividing tumours. We show that PRMT6 dependent repression of the p21 promoter is independent of the p53 status. Although p21 is a direct p53 target (28,29), it is known to be regulated by many other tumour suppressors, such as BRCA1 (30), CHK2 (31), FOXP3 (32), transforming growth factor-β (33) and importantly repressed by oncogenes, such as c-myc (34) and Gfi1 (35). It remains to be determined whether alterations in these genes can render cells refractory to the growth inhibition because of PRMT6 inactivation and subsequent p21 upregulation.

PRMT6 is not known to bind DNA directly. The identification of the mechanism recruiting PRMT6 to the p21 promoter is an aspect that is worth to investigate in the future. Myc, is a possible candidate, as it is known to directly repress p21 through interacting with Miz-1 (34,36), and given that PRMT6 is a direct c-myc target (37), it could be part of a feed forward loop propelling cell growth in tumour cells. Alternatively, repression by PRMT6 could be associated with the Polycomb group proteins (PRC2), as we do observe the presence of H3K27me3 at the p21 promoter (Figure 3D). The positive correlation between H3R2me2a and H3K27me3 has been observed previously at the biochemical level (6) and on several repressed promoters (23), but how PRMT6, and PRC2 itself, is recruited to chromatin in mammalian cells remains an open question (38).

To conclude, our study points at PRMT6 as an attractive target for cancer drug therapy, suggesting that PRMT6 inhibitors should be tested preferentially in selected subgroups of cancer that have fewer therapeutic alternatives due, for example, to an inactive p53 pathway and low expression of p21.

## SUPPLEMENTARY DATA

Supplementary Data are available at NAR Online: Supplementary Figures 1–6 and Supplementary Methods.

## FUNDING

Funding for open access charge: Biomedical Research Council of A*STAR (Agency for Science, Technology and Research), Singapore.

*Conflict of interest statement*. None declared.

## Supplementary Material

Supplementary Data
