# Supplementary material for: p53-Independent regulation of p21Waf1/Cip1 expression and senescence by PRMT6
Source: Nucleic Acids Res. 2012 Sep 16;40(19):9534–42. doi: 10.1093/nar/gks858 (PMC3479215; doi:10.1093/nar/gks858)
Supplement: Supplementary Data [file supp_40_19_9534__index.html]

p53-Independent regulation of p21Waf1/Cip1 expression and senescence by PRMT6 — p53-Independent regulation of p21Waf1/Cip1 expression and senescence by PRMT6 — Supplementary Data 

# p53-Independent regulation of p21Waf1/Cip1 expression and senescence by PRMT6

## Supplementary Data

files

**Files in this Data Supplement:**

- Supplementary Data - pdf file
