# Supplementary material for: p53-Independent regulation of p21Waf1/Cip1 expression and senescence by PRMT6
Source: Nucleic Acids Res. 2012 Sep 16;40(19):9534–42. doi: 10.1093/nar/gks858 (PMC3479215; doi:10.1093/nar/gks858)
Supplement: Supplementary Data [file supp_gks858_nar-01650-x-2012-File007.pdf]

PRMT 6 Over Expressed cell line **PRMT 6 Knock Down cell line**

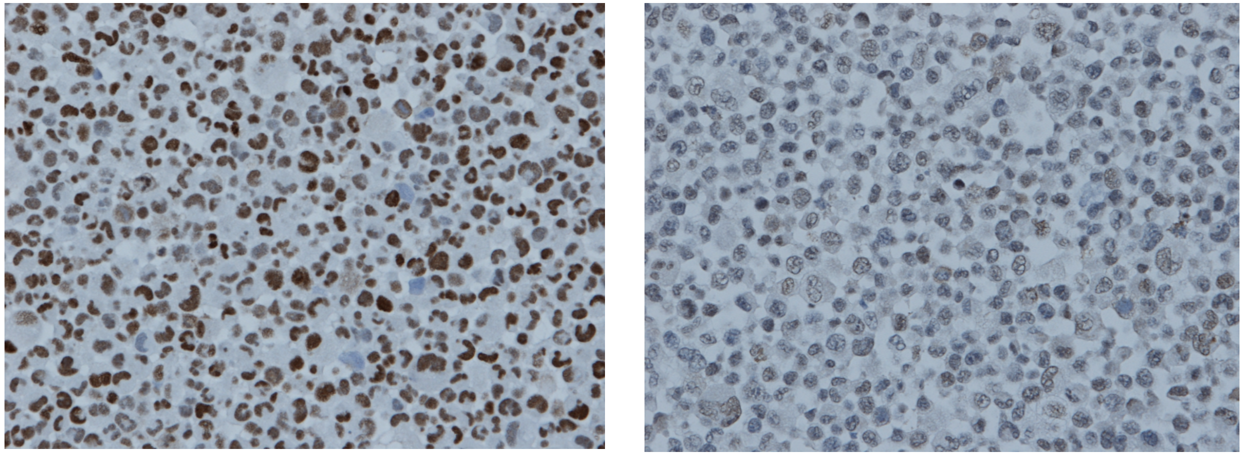

Supplementary Figure 1. Validation of the specificity of the rabbit polyclonal anti-PRMT6 antibody raised in house.

Primary antibody recommended dilution: 1:5000

Epitope Retrieval: Citrate buffer 10mM pH6, Pressure cooker (121°C) 45 min.

Peroxidase block: 3% hydrogen peroxide 30 min.

Serum Block: 10% Goat serum in TBST 60min.

Primary Antibody incubation: 60min. Room Temperature

Secondary Antibody: DAKO Anti Rabbit HRP Polymer. Cat No. K4003

Dilution: Undiluted

Incubation Time: 30 min.

DAB Kit: DAKO Cat No. K3468

Localization: Nuclear

Supplementary Fig.1

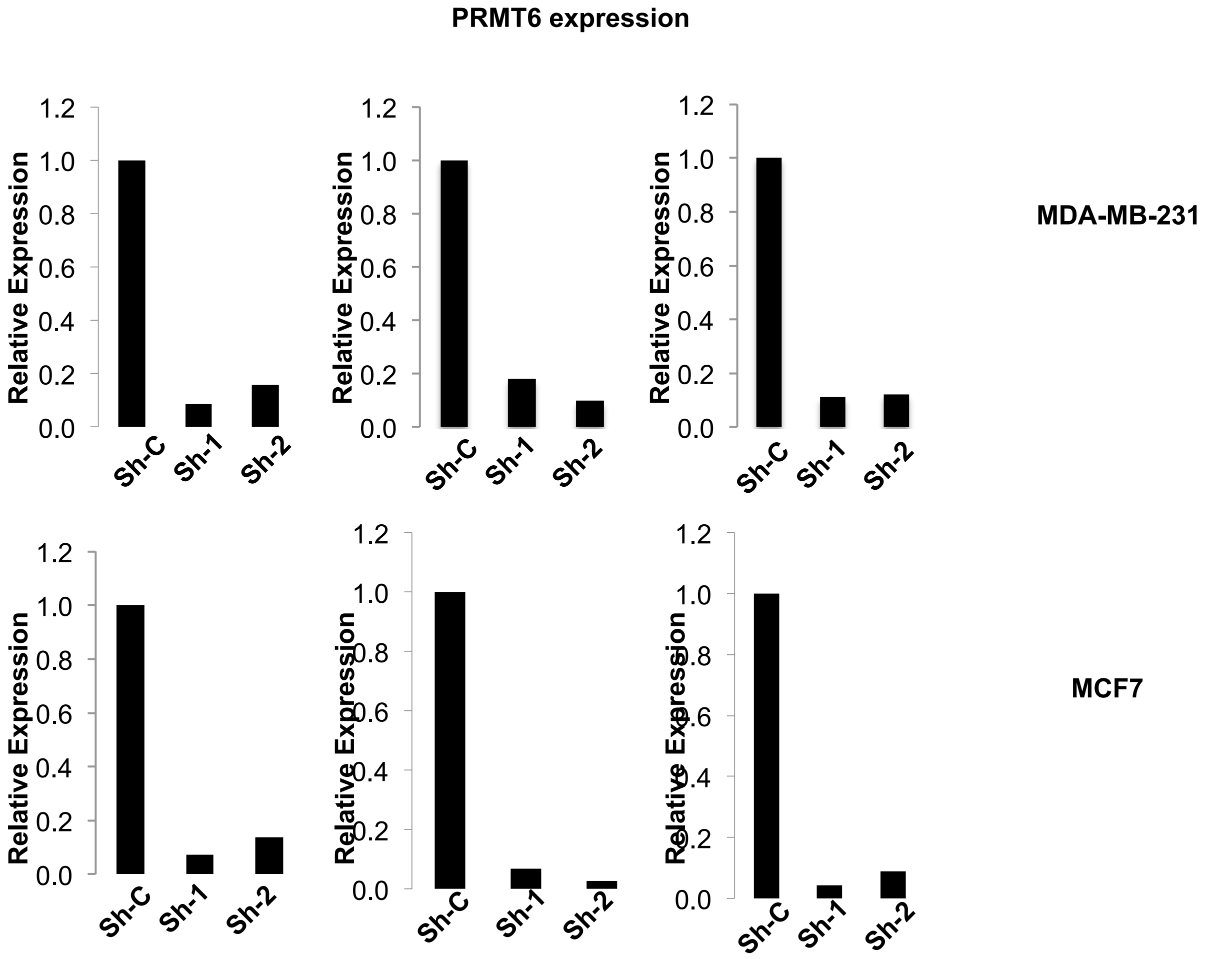

Supplementary Figure 2. Depletion of PRMT6 RNA using 2 independent sh-RNA constructs (sh-1 and sh-2) in MCF7 (upper panel) and MDA-MB-231 (lower panel). Three independent experiments are shown. The values were normalized relative to Actin and the scramble control (sh-c) cells.

Supplementary Fig.2

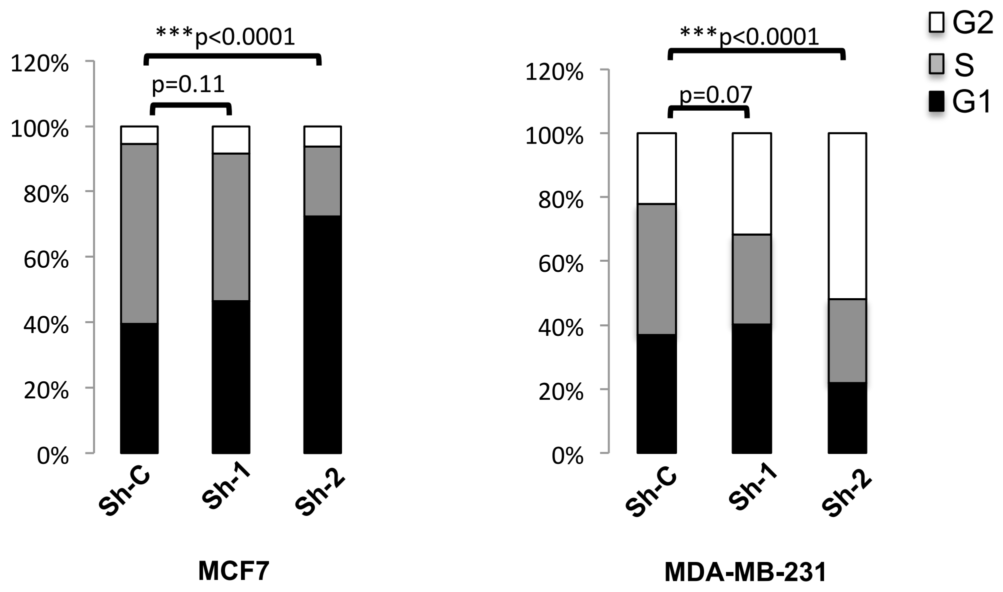

Supplementary Figure 3. Cell Cycle profiling of PRMT6 depleted (sh-1 and sh-2) or control (sh-c) in MCF7 (left panel) and MDA-MB-231 (right panel) using FACS analysis. p-values are indicated.

Supplementary Fig.3

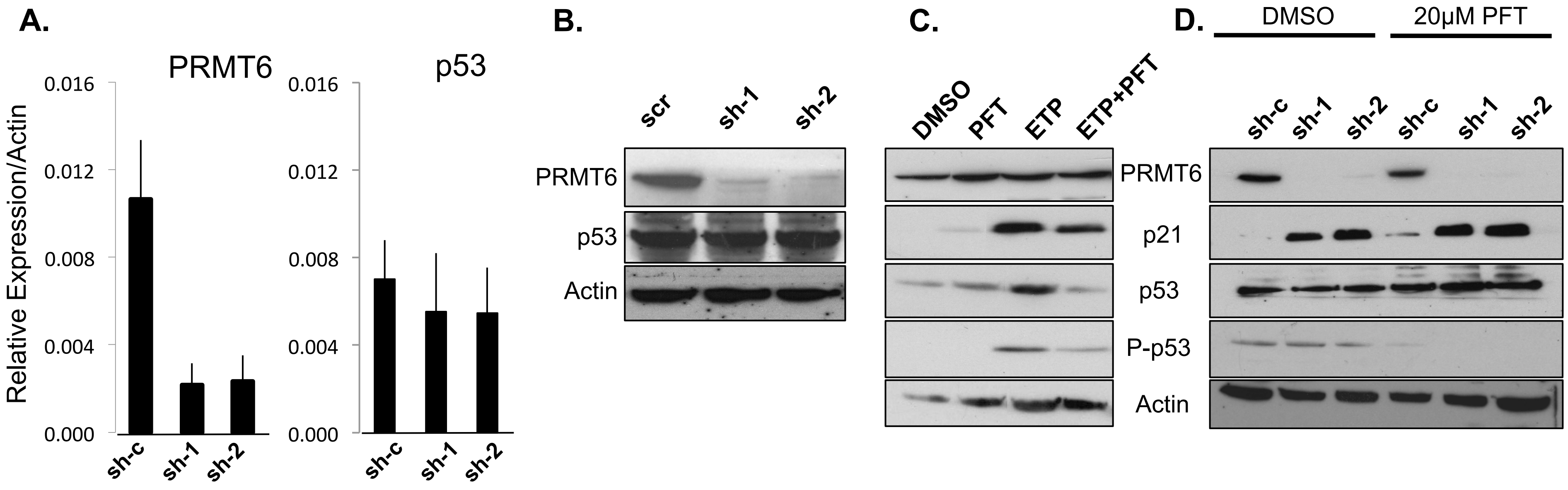

Supplementary Figure 4. PRMT6 mediated induction of p21 is independent of p53. **A.** PRMT6 depletion in MCF7 does not lead to any significant changes in p53 expression at both RNA or **B.** protein levels. **C.** Treatment of MCF7 cells with Pifithrin-α (PFT, 20μM), Etoposide (ETP, 20μM) or both (PFT+ETP) or control vehicle (DMSO), as indicated. ETP treatment alone induced phosphorylation and stabilization of p53 as well as p21 upregulation while ETP treatment on PFT pretreated cells reduced phosphorylated p53 and p21 upregulation. Actin was used as loading control. **D.** MCF7 cells were pretreated with PFT or control vehicle (DMSO) followed by PRMT6 KD (sh-1 or sh-2). PRMT6, p21 and p53 levels are shown and do not change irrespective of p53 inhibition (PFT). Actin was used as loading control.

Supplementary Fig.4

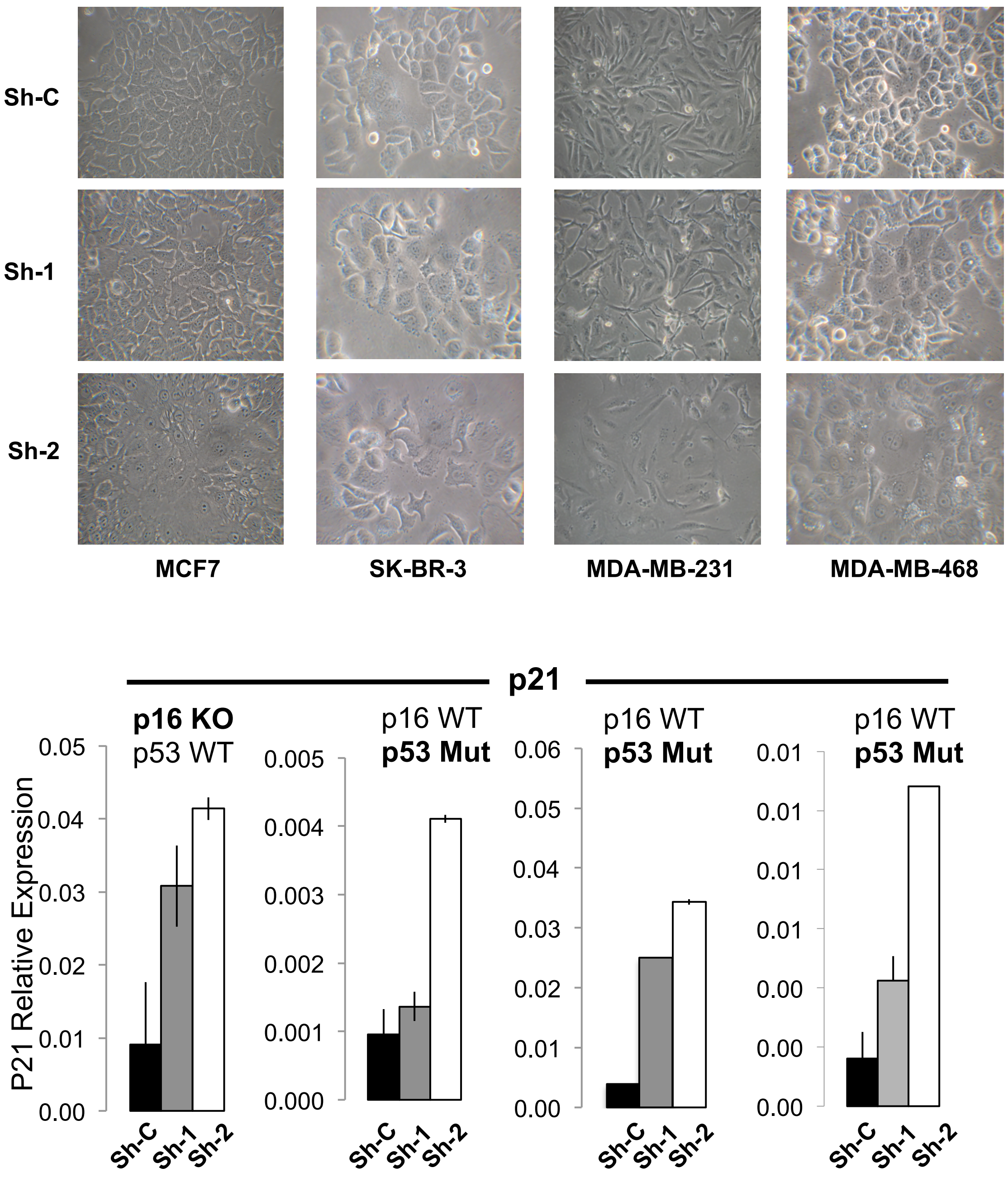

Supplementary Fig.5

Supplementary Figure 5.
PRMT6 depletion lead to senescence-like morphological changes and growth arrest in different cancer cell lines (upper panel) along with consistent p21 upregulation (lower panel) irrespective of the p53 or p16 status (indicated in the bottom panel for each cell line).

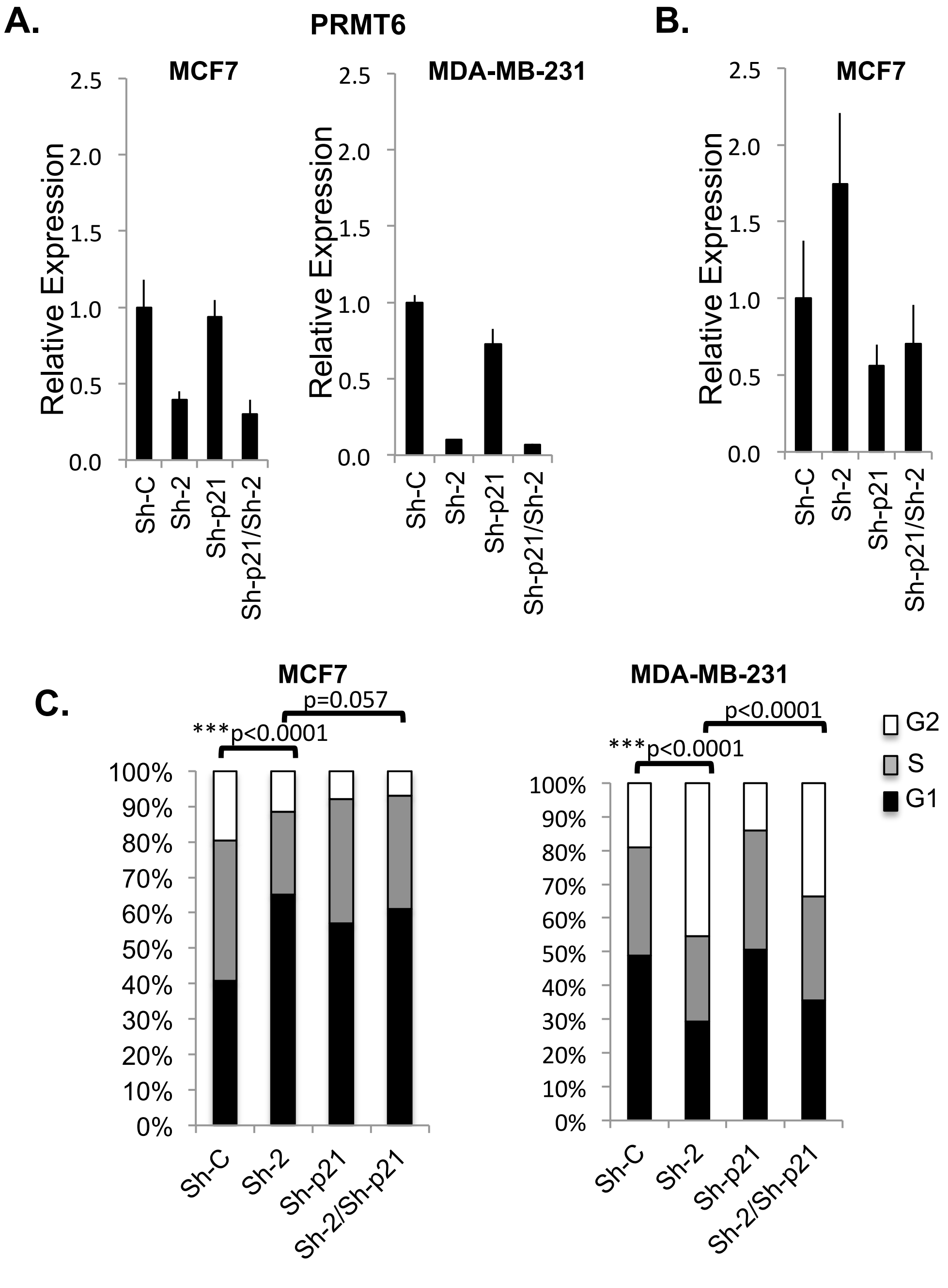

Supplementary Figure 6. A. PRMT6 levels are not significantly affeted by p21 kd in both the MCF7 and MDA-MB-231 cell lines. **B.** sh-p21 leads up to 40-50% depletion of the p21 RNA. **C.** Cell cycle profile of MCF7 and MDA-MB-231 cells depleted for p21 and/or PRMT6 by FACS profile. p-values are indicated.

Supplementary Fig.6

Supplementary Methods:

FACS analysis:

Actively growing MCF7 or MDA-MB-231 cells with different conditions were washed with PBS, trypsinized and then fixed with 70% Ethanol. Fixed cells were then rehydrated by serial washes with PBS and then incubated with 250 ug/ml RNAase and 54uM Propidium Iodide for 1-4 hrs at room temperature. Percentage of different cell populations were recorded in a Becton and Dickinsons FACS calibur and reproducible and representative data sets are shown.

Etoposide and Pifithrin Treatment:

MCF7 cells were treated with 20μM Pifithin (PFT, Sigma, Cat No# P4359) at least for 2 hrs before infection with PRMT6 KD viruses. PFT was again added to the cells during selection. Cells were harvested 72 hrs post infection for protein analysis. For p53 activation MCF7 cells were treated with 20μM Etoposide (ETP, Sigma, Cat No# E1383) for 20 hrs with or without 20μM PFT pretreatment. Cells were harvested after 20 hrs for protein analysis, actin was used as loading control.
